# *Lachnospiraceae* shift in the microbial community of mice faecal sample effects on water immersion restraint stress

**DOI:** 10.1186/s13568-017-0383-4

**Published:** 2017-04-17

**Authors:** Shiyin Li, Zelin Wang, Yun Yang, Sha Yang, Chenchen Yao, Kaiyun Liu, Sixin Cui, Quanming Zou, Hongwu Sun, Gang Guo

**Affiliations:** 1 National Engineering Research Center of Immunological Products, Department of Microbiology and Biochemical Pharmacy, College of Pharmacy, Third Military Medical University of PLA, 30 Gaotanyan Street, Shapingba District, Chongqing, 400038 People’s Republic of China; 2 Department of Epidemic Prevention, Hospital of Troop 66325, Chinese PLA, Beijing, 102202 People’s Republic of China

**Keywords:** Water immersion restraint stress, Faecal microbiota, 16s rRNA, Microbial community, Illumina Miseq sequencing

## Abstract

Stress, including both psychological and physical stimulation, can cause changes in the microbiota and mucosal function of the gastrointestinal system. There are few research studies available about the faecal microbiota changes after stress, such as water immersion restraint stress (WIRS). Therefore, in this study, we focused on analysing the composition changes of faecal microbiota in WIRS mice. The WIRS model, in which Blab/c mice were immersed in 21 ± 2 °C water for 4 h each day for 14 days, was established. Behavioural changes, the serum levels of corticosterone, IFN-γ and IL-17 and gastric mucosal injury were also assessed. Ten faecal microbiota samples were detected by Illumina Miseq sequencing of the 16S rRNA genes from 367205 characterised sequences. Finally, we find significant differences in the faecal microbiota composition between the control and the WIRS groups. There was an obvious increase in *Lachnospiraceae* in the WIRS mice (p = 0.0286, p < 0.05), which is associated with human diseases, such as ulcerative colitis, Crohn’s and celiac disease. Our research indicates that stress changes in the faecal microbiota. These results suggest that observing shifts of the intestinal microbiota is a promising method to explore the mechanism of the stress associated with gastrointestinal diseases and to provide us with a better understanding of the relationship between the microbiota and disease.

**Electronic supplementary material:**

The online version of this article (doi:10.1186/s13568-017-0383-4) contains supplementary material, which is available to authorized users.

## Introduction

Microbiota is indispensable for human health, but the mechanism of how this system influences the host is still unclear (Bian et al. 2013). Once the composition balance of the intestinal microbiota is broken, it leads to a variety of diseases, such as irritable bowel syndrome, metabolic disorders, atherosclerosis and inflammatory bowel disease. Recent studies indicate that the changes of intestinal flora were closely associated with diabetes, obesity, hyperlipidemia, cardiovascular disease, colon cancer and other intestinal diseases. Modulation of the microbiota composition provides an opportunity to promote or recover health (Kallus and Brandt 2012). The gut microbiota status is closely related to many factors, such as emotional and dietary and immune dysfunction (Bian et al. 2013). Stress is a state of mental or emotional strain, which causes different physiological or pathological changes depending on its type and severity. There are various types of stress, including immobilization/restraint stress, water immersion restraint stress and cold stress. Water immersion restraint stress (WIRS) is one of the most widely used models to discover gastric mucosal damage in animals (Jaggi et al. 2011) Stress response occurs via the hypothalamic–pituitary–adrenal axis and the sympathetic-adrenal system (Ohta et al. 2009). Serum levels of CORT, IL-17 and IFN-γ increase after stress treatment (Blossom et al. 2016; Guo et al. 2009; Peerzada et al. 2016). Studies show that stress causes gastrointestinal disorders, such as irritable bowel syndrome (Ohta et al. 2009). This disorder is related to changes of the intestinal flora composition. Stress may be an important factor that induces activity and composition changes in the gut microbiota, especially in some specific bacterial species of microorganisms. Until now, there are few reports about faecal microbiota changes due to stress. Therefore, we want to study whether faecal microbiota changes are impacted by stress. In this study, a stress model of WIRS was established, and we evaluated changes in behaviour, the serum levels of CORT, IFN-γ and IL-17 and gastric mucosal injury. The faecal microbiota changes in relation to stress were detected by Miseq sequencing analysis of 16S rRNA genes. Finally, we analysed the differences in the faecal microbiota composition between normal and WIRS groups. These faecal microbiota changes may help to explore the mechanism of stress associated with gastrointestinal diseases and provide better suggestions of the relationship between the microbiota and disease.

## Materials and methods

### Ethics statement

Our study was approved by Third Military Medical University Animal Ethics Committee (Project Number: 201104), Chongqing, China, according to the principles of Helsinki Declaration. The protocol for early/humane endpoints in cases where animals became severely ill prior to the experimental endpoint was implemented according to the animal facility rules. If the animals are moribund or in a state of impending death, they should be immediately euthanized. The animals were monitored daily, and none mouse neither died nor exhibited clinical signs of suffering, distress or pain during the experimental procedure.

### Animal and animal treatment

Ten specific-pathogen-free Blab/c mice (6-week-old females with five in the normal group and five in the stress group) were purchased from the Centre of Experimental Animals, Third Military Medical University of Chinese PLA (Chongqing, China). All the mice were housed and fed an autoclaved pellet diet and sterile water under the following conditions: five mice per cage; 23 °C; humidity 50%; half-day periods of light or dark; and quiet surroundings with minimal disturbance. In order to keep a consistent flora environment, all the mice were collected into one cage from 3 to 5 weeks after birth. Then, all the mice were used in the experiments after a 1-week adaptation.

### The establishment and assessments of the WIRS model

Referring to a previously described method (Ohta et al. 2009) and mice number (Hsiao et al. 2014), the establishment and assessment of the WIRS model was performed based on the preliminary experiments. Briefly, the limbs and belly of the stress group mice were bound to foam boards to restrict their activity. Then, all the mice were immersed vertically 4 h every day in a 21 ± 2 °C water bath cages. The water level was kept to the sternum xiphoid level of the mice, and this continued for 14 days. The stress behaviour of all the mice was observed and recorded, and the level of serum CORT, IL-17 and IFN-γ were measured at 1 and 2 weeks of the WIRS. The gastric mucosal injuries of all the mice were also observed after HE dyed,

### DNA extraction and PCR amplification of faecal specimens

According to the experiment protocol (Ohta et al. 2009), microbial DNA was extracted from faecal samples of all mice and used Tiangen faecal DNA Purification Kit (Tiangen, China) according to manufacturer’s protocols. The primers for 338F 5′-(barcode-ACTCCTACGGGAGGCAGCA)-3′ and 806R 5′-(GGACTACHVGGGTWTCTAAT)-3′ were synthesized by Sangon Biotech (Shanghai, China). Then, via the PCR system (4 µL of 5× Fast Pfu buffer, 2 µL of 2.5 mM dNTPs, 0.8 µL of each primer (5 μM), 0.4 µL of Fast Pfu Polymerase, and 10 ng of DNA by polymerase and faeces, the thermal profile of the PCR for the 16S ribosomal DNA gene region V3–V4 was as follows: 95 °C for 2 min; 95 °C for 30 s; 55 °C for 30 s; 72 °C 45 s; and 30 cycles and a final extension of 72 °C for 5 min.

### MiSeq pyrosequencing

The amplicons were extracted and purified from a 1% agarose gel in accordance with the manufacturer’s instructions using a AxyPrep DNA Gel Extraction Kit (Axygen Biosciences company, Union City’s, CA, USA) and using Quanti Fluor™-ST (Promega, USA) for quantification based on the previous describe methods (Zheng et al. 2016). Uqimolar purified amplicons were combined and paired-end sequenced according to standard protocols (2 × 250) of the Illumina MiSeq platform (Yan et al. 2015). The original read is stored in the NCBI sequence reads archive (SRA) database (Accession Number: SRP050150).

### Bioinformational and statistical analysis of the sequencing

Raw fastq files were demultiplexed, quality-filtered using QIIME (version 1.9.1) with the following criteria: (1) The 300 bp reads were truncated at any site receiving an average quality score <20 over a 50 bp sliding window, discarding the truncated reads that were shorter than 50 bp. (2) exact barcode matching, two nucleotide mismatch in primer matching, reads containing ambiguous characters were removed. (3) Only sequences that overlap longer than 10 bp were assembled according to their overlap sequence. Reads which could not be assembled were discarded (Amato et al. 2013). The operation unit (OTU) with 97% similarity and clustering cut off the Chimerical sequences was used to identify and exclude with UPARSE (7.1Version http://drive5.com/uparse/). Each system of 16S rRNA gene sequences developmental affiliation was analysed by RDP. Classification (http://rdp.cme.msu.edu/) was performed using a confidence threshold for Silva (SSU115) at 70% of the 16S rRNA database.

### Statistical analysis

All values are expressed as mean ± SEM (n = 5). Differences between two groups were tested using Student’s *t* test. All data analyzed using GraphPad Prism 5.0 software (San Diego, CA, USA). Significant differences are expressed as *p < 0.05, **p < 0.01, and ***p < 0.001.

## Results

### The establishment and assessment of the WIRS mice model

**Fig. 1:**
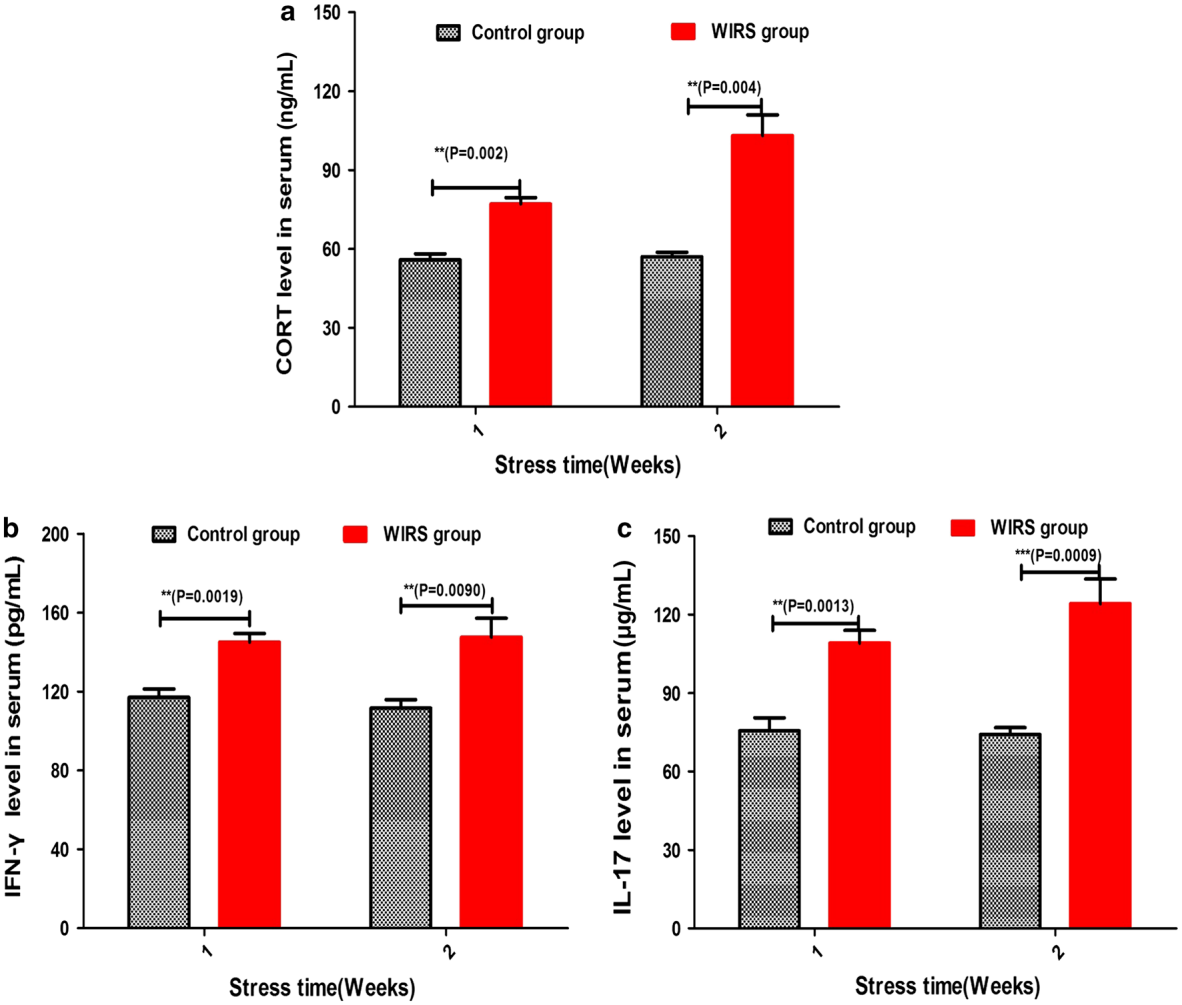
Behaviour and biochemistry results of the WRIS. **a** CORT levels in serum; **b** IFN-γ levels in serum; **c** IL-17 levels in serum. Data in **a**, **b**, and **c** are expressed as the mean ± SEM (n = 5). *P < 0.05; **P < 0.01; ***P < 0.001

The WIRS model was established and assessed by the abnormal indices, including behaviour, pathology, and the level of corticosterone, IFN-γ and IL-17 in the serum after 2 weeks between the control and the WIRS group. Compared with the behaviour of the normal group, the stressed mice huddled in the corner of the cage, screamed, had an accelerated heartbeat, and closed their eyes silently. The stressed mice also had an obvious reduced weight, and their faecal colour turned to a yellow-green. CORT levels in the serum increased at 1 week of stress and further increased at 2 weeks as shown in the Fig. 1a. Therefore, CORT levels were significantly higher in the mice exposed to stress compared to the normal group in 2 and 4 weeks (p = 0.002, p < 0.01; p = 0.004, p < 0.01). Similar results were shown for the IFN-γ levels, which increased from 1 to 2 weeks in the stressed group as shown in the Fig. 1b. The IFN-γ levels in the serum showed obvious differences between the normal mice and the stress groups in 2 and 4 weeks (p = 0.0019, p < 0.01; p = 0.004, p < 0.01). In addition, the level of IL-17 in the serum, as demonstrated in the Fig. 1c, increased with stress and showed obvious differences compared with the normal group (p = 0.0013, p < 0.01; p = 0.009, p < 0.01). We observed that there are more inflammatory cell infiltrations of stress group (Additional file 1: Figure S1A) in the gastric mucosal tissue than normal group (Additional file 1: Figure S1B). In short, a long-term WIRS model was successfully established as demonstrated by the series changes that were observed, including the behavioural, serum corticosterone, IFN-γ and IL-17 levels and gastric mucosal injury changes.

### Sample collecting and DNA OTUs assessments

Two weeks after the end of the WIRS, we took the same parts of faecal samples for bacterial genome extraction and detected the Blab/c mice intestinal flora colonisation using Miseq methods. A total of 349,596 reads and 420 OTUs were obtained from the ten samples through the 16s rRNA Miseq. The stress group (contained 32,742–42,261 reads, with different OTUs ranging from 148 to 191) showed no obviously differences (p = 0.1474, p > 0.05; p = 0.5368, p > 0.05) compared with the control group (contained 29,176–33,304 reads and had OTUS ranging from 153 to 192). In an effort to determine whether all the OTUs presented in the data set were recovered in the Miseq study, more analyses were performed.

### Alpha-diversity

In this study, the alpha diversity (represented the richness and diversity of the sample type) of three metrics, including the Chao metric (represented the species richness), the Species metric (simply represented the count of unique OTUs) and the Shannon index (represented the species diversity), were analysed. Results of the estimators table are shown in Table 1.

**Table 1:** Table of alpha-diversity analysis

Sample ID	Reads	OTU	ace	Chao	Shannon	Simpson
Control.1	33,304	168	174 (170, 186)	172 (169, 185)	2.97 (2.95, 3)	0.1746 (0.1707, 0.1785)
Control.2	30,864	153	165 (158, 183)	164 (156, 189)	3.52 (3.51, 3.54)	0.0532 (0.0522, 0.0542)
Control.3	39,772	182	195 (187, 214)	230 (197, 333)	2.7 (2.67, 2.72)	0.2657 (0.2608, 0.2705)
Control.4	29,176	184	192 (187, 207)	204 (190, 250)	3.57 (3.55, 3.59)	0.0618 (0.0605, 0.0632)
Control.5	31,430	192	200 (195, 213)	200 (194, 221)	3.67 (3.65, 3.69)	0.0604 (0.0589, 0.062)
Stress.1	32,742	155	168 (160, 187)	166 (159, 187)	3.3 (3.28, 3.32)	0.082 (0.0802, 0.0837)
Stress.2	42,261	163	173 (167, 189)	173 (166, 195)	2.95 (2.93, 2.96)	0.1713 (0.1678, 0.1748)
Stress.3	37,102	149	159 (153, 174)	157 (151, 177)	2.73 (2.71, 2.75)	0.1943 (0.1904, 0.1982)
Stress.4	33,570	186	198 (191, 216)	197 (190, 220)	3.5 (3.48, 3.52)	0.0723 (0.0705, 0.074)
Stress.5	39,375	191	208 (198, 230)	226 (203, 287)	3.3 (3.29, 3.32)	0.1044 (0.1021, 0.1068)

Data are expressed as the mean ± SEM (n = 5)

* p < 0.05

** p < 0.01

*** p < 0.001

At the species level, a maximum number of 226 operational taxonomic units were recorded from the ten faecal samples, and 183 and 194 operational taxonomic units were recorded for the stress and the control samples, respectively. The Simpson diversity index and the richness (ACE) values were higher in the stress samples than the control at all levels of computing systems development (p = 0.7362, p > 0.05). In addition, the Shannon index was lower than the control group sample. The Shannon diversity index number was obtained by the OTU and the Simpson index of diversity of the non-parametric estimation (chao) and abundance (ACE) values did not show a similar pattern (p = 0.5697, p > 0.05). Because of the unique sequence analysis, the species diversity, the Shannon index (3.56 in the normal group, 3.44 in the WIRS group) and the Simpson index (0.09 in the normal group play against 0.0962 in the WIRS group) were evaluated. Thus, the overall level of bacterial diversity between the WIRS group and the control was not different (p = 0.5961, p > 0.05; p = 0.9727, p > 0.05 for the two groups, Shannon and Simpson index). In addition, the bacterial communities between the two groups were not different. For both groups, at a 3% non-similarity analysis of the faecal bacteria community, the detection number of the OTU boards and ACE diversity index estimated that the total number of the proposed additional evidence of the natural communities that were sequenced was well covered. The identification of 367,205 samples/sequences (minimum sampling depth) by the alpha diversity analysis was sparse. This indicates that, at the level of coverage, the 16S rRNA sequences identified in these groups represent the majority of the study sample bacterial sequences.

### The curve analysis of rarefaction, Shannon, Specaccum accumulation and relative abundance

**Fig. 2:**
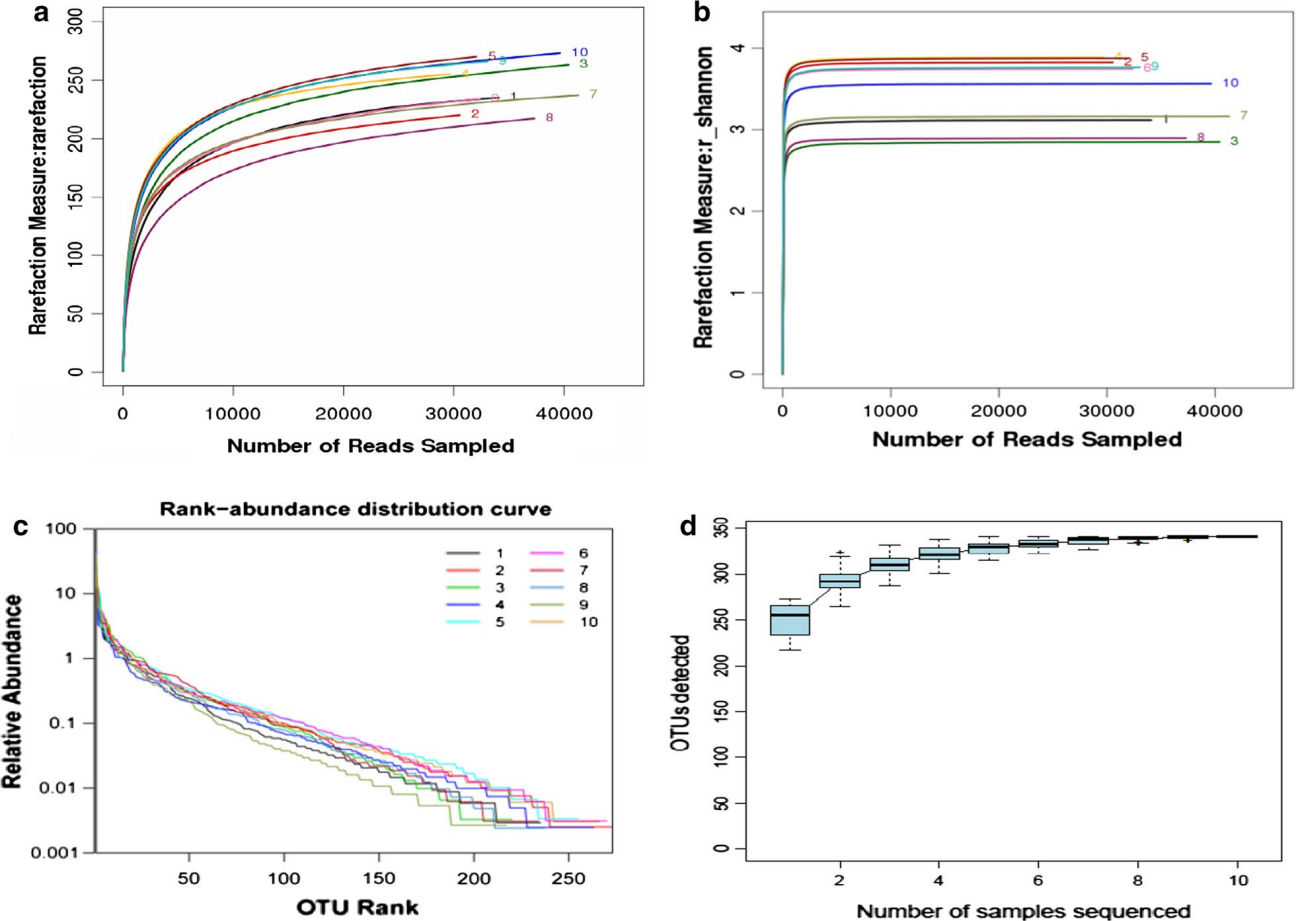
Results of the rarefaction, Shannon, Specaccum accumulation and relative abundance analysis. **a** The rarefaction curve, **b** Shannon curve, **c** Specaccum accumulation curve, and **d** relative abundance curve

The rarefaction and Shannon analysis of the bacterial communities derived from the all mice are shown in the Fig. 2a, b. And also, the rarefaction and Shannon analysis of WIRS and control samples are shown in Additional file 1: Figures S2, S3. There was no obvious difference in the rarefaction curve trend and the Shannon curve between the WIRS and control groups (p = 0.0513, p > 0.05; p = 0.0524, p > 0.05). The rarefaction and Shannon curve of the faecal samples reached saturation at the level of species, genera, and family even after the retrieval of more than 5000 sequences. And also, Specaccum accumulation curve and the relative abundance curve analysis of all mice are shown in the Fig. 2c, d. Further analyzed and found that Specaccum accumulation curve and the relative abundance curve analysis of the WIRS and control samples were dissimilar as shown in Additional file 1: Figures S4, S5. We found that the Specaccum accumulation and the relative abundance curves were not different between the WIRS and control samples (p = 0.0542, p > 0.05; p = 0.0563, p > 0.05). In short, the Shannon diversity index (Fig. 2a), the nonparametric estimation of diversity (Fig. 2b) (Chao), the abundance (ACE), the rarefaction curves (Fig. 2c) and the specaccum cumulative curve (Fig. 2d) obtained showed a similar pattern by the number of OTU boards and the Simpson index. In the Fig. 2c, curve shows that the in the number of species detected increase with sequencing depth increase. But to a certain level, there is not many species increased with increase the amount of sequencing. This is to say that curve has reached a plateau will. And also, it has shown that dilution curve flattens has sequencing quantity enough. In the Fig. 2d, cumulative curve show that species number will increase with samples. Similar, the number range of the sample hasn’t s increased the final trend. The relatively reasonable sampling number can reflect the class of all species in the sample. Four saturation curve exhibits the saturation curve, indicating that the measurement at the gate level covers almost the full extent of the genetic diversity; further, we need more sequencing at the family, genus and species level. Thus, the data from the control and the WIRS group can be used in further analysis.

### Taxonomic composition change of stress

**Fig. 3:**
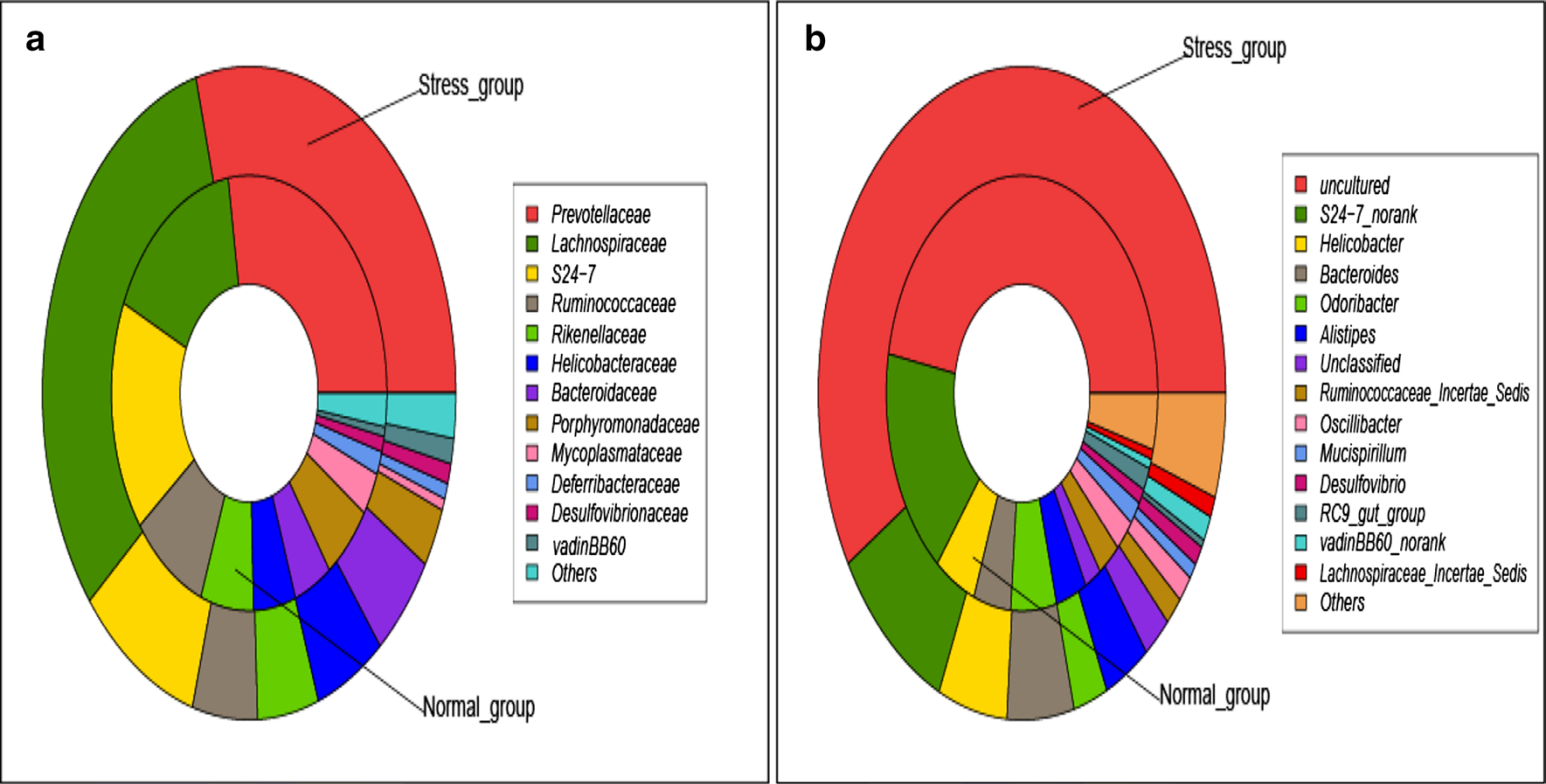
Taxonomic composition. **a** The taxonomic composition diagram at the family level, and **b** taxonomic composition diagram at the genus level

Ten samples, which were comprised of different numbers abundances of OTUs could not be classified into two groups, were assigned as a control and stress group. The faecal bacterial OTUs were divided into 13 families or 37 genera. The taxonomic composition data at the level of family showed that a vast majority of sequences in five major phyla, including *Odoribacter, Helicobacter, Alistipes, S24*-*7_norank* and *Bacteroides,* as shown in the Fig. 3a. Of these major phyla, *Odoribacter* was the most dominant phyla in the faecal microbiota of the normal group, while bacteria belonging to *Helicobacter, Bacteroidetes, Alistipes* and *S24*-*7_norank* constituted the complex faecal microbiota in the WIRS group. In addition, the taxonomic composition at the level of genus showed that four major phyla, including *Lachnospiraceae, S24*-*7, Ruminococcaceae, and Bacteroiaceae,* as shown in the Fig. 3b.

### Venn diagram

**Fig. 4:**
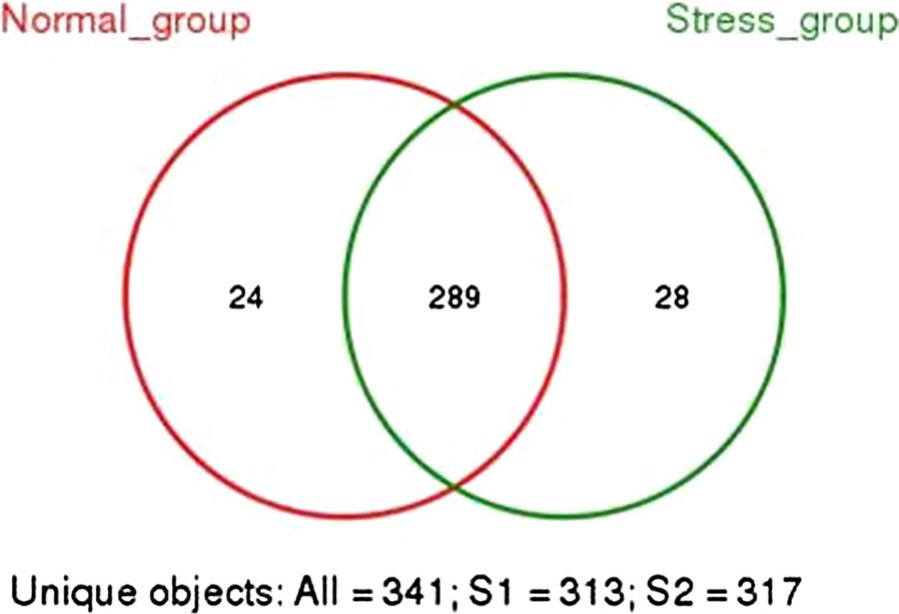
Venn diagrams for overlap between normal versus stress. Venn diagrams show the overlap in all OTUs calculated at the 3% dissimilarity level. The number of species in normal and stress group are 313 and 317. The number of species shared between groups is 289. Percentage of species that are shared in stress and normal groups is 84.75%. Venn diagrams show the overlap in all OTUs calculated at the 3% dissimilarity level. The number of species in normal and stress group are 313 and 317. The number of species shared between groups is 289. Percentage of species that are shared in stress and normal groups is 84.75%

For an overview comparison between the different sample types, we merged the results found in the different faecal communities and displayed the overlapping genera within stress and normal groups in the Venn diagram as shown in Fig. 4. The sequences are from the normal group represented 24 different genera (313 genera), with 28 different genera in the WIRS group (317 genera) and 289 of the same genera in the control group.

### Heat map analysis

**Fig. 5:**
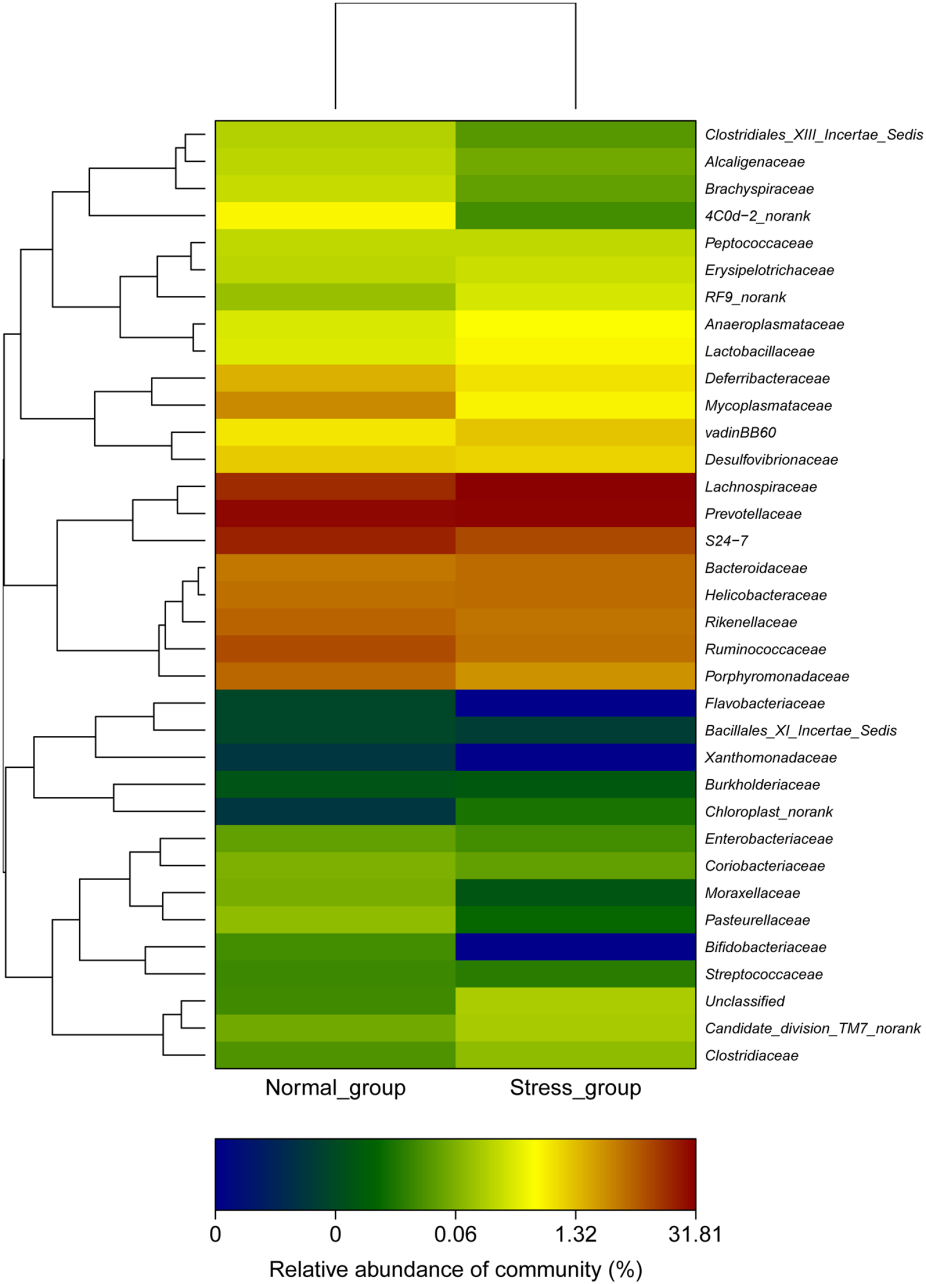
Heat map diagram at the family level

**Fig. 6:**
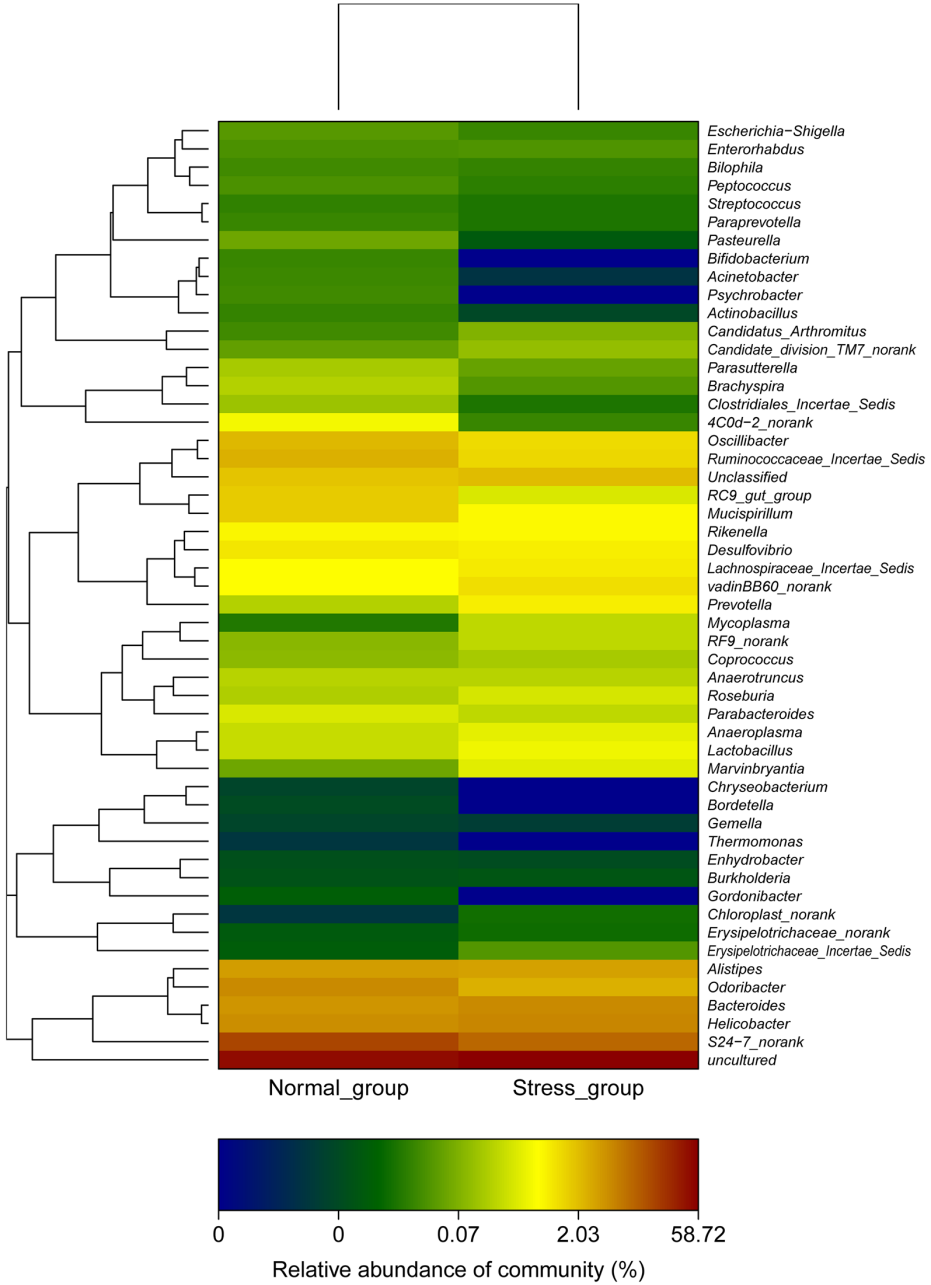
Heat map diagram at the genus level

The heat map of the bacteria composition from all mice is shown in the Additional file 1: Figures S6, S7 at the family and genus level. Study showed that there is an obvious increase in *Helicobacter, Bacteroides, Alistipe*s, and *Vandin BB60*-*_norank* at the family level after stress in the Fig. 5. In these ratios of bacteria, the *Helicobacter* and *Bacteroides* increase dominated the bacterium after stress as shown in the Fig. 5. The heat map of the genus represented in Fig. 6 shows that the individuals within a same family cluster together when the genus level distribution of the gut flora is considered. The *Lachnospiraceae, Helicobacter, and Bacteroides* increased in the stress compared with the normal group. In addition, *Prevotellaceae* dominated in the normal and stress groups. A comparison of our reports suggests that the modification of the stress treatment may depend on the genetic background of the host animals, the baseline structure of the microbiota and the stress treatment protocols employed.

### Community analysis

**Fig. 7:**
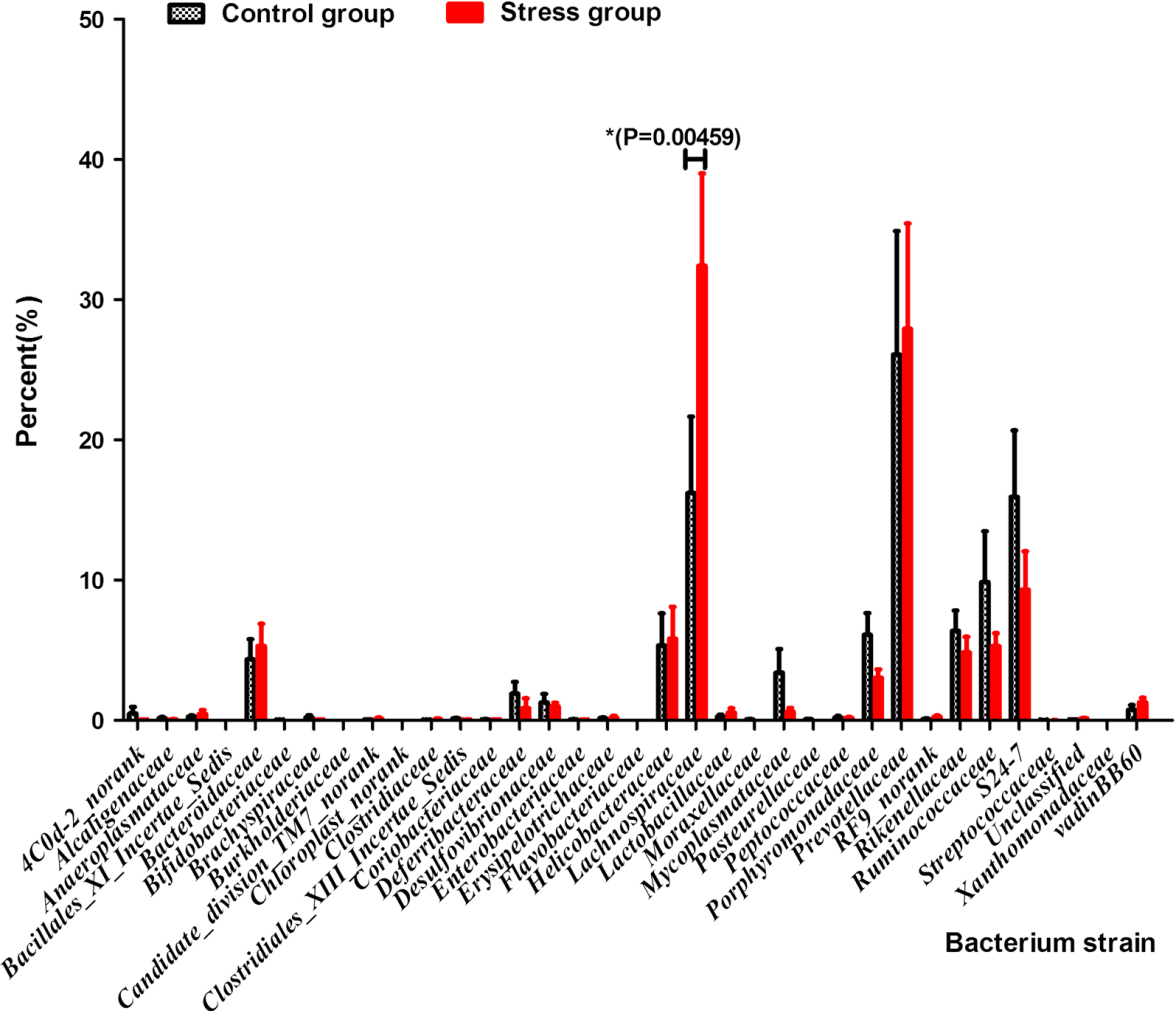
Figure of community analysis. Data are expressed as the mean ± SEM (n = 5). *P < 0.05; **P < 0.01; ***P < 0.001

Bacterial differences between the WIRS and the normal group were observed by the 16S rRNA encoding genes present in the faecal pellets. An analysis of the gut microbiota of the WIRS mice showed large variations in the bacterial composition during treatment as shown in the Fig. 7. The bacterial composition of the stress group was dominated by *Lachnospiraceae, Prevotellaceae, S24*-*7* and *Ruminococcaceae*. The most striking shift occurred in the *Lachnospiraceae* genus whose frequency increased at the beginning of the treatment to 12.22 and 36.36% over the stress course. In addition, *S24*-*7* and *Ruminococcaceae*, reduced at the beginning of the stress treatment from 16.85 and 9.47% to 9.68 and 5.15%, respectively. Compared with the control group, the stress group had a very unusual gut microbiota that was dominated by *Lachnospiraceae* (36.36%) and *Prevotellaceae* (25.59%), but its abundance decreased dramatically reaching the lowest values during the broad-spectrum stress treatment. After the stress treatment, the abundance of the microbial community changed again, in which *Lachnospiraceae* was predominant. There is a significant difference between the stress and the normal control groups (p = 0.0286, p < 0.05). The community profile of the microbiota at the faecal showed a significant difference between two groups.

## Discussion

Plenty of microbiome studies are defining the structure and function of the gut microbiome in health and active disease states. The intestinal microbiota interacts extensively with the host through metabolic exchanges and the co-metabolism of substrates to maintain the normal function and health status of the intestinal tract and the whole body (Rooks et al. 2014) Increasing research indicates that changes of the intestinal flora are closely associated with diabetes, obesity, hyperlipidemia, cardiovascular disease, colon cancer and other intestinal diseases. Thus, in this study, we want to know that how the faecal microbiota changes after stress treatment.

Stress causes distinctive pathological changes via physiological and psychological approaches (Mohd Fahami et al. 2012). Various levels of stress cause different injuries or diseases including diabetes, hypertension, and gastric ulcers. Stress mainly affects the stomach, and stress-induced gastric ulceration is the most common disease (Rooks et al. 2014). The gut function and the microbiota are influenced by stress, which occur via mucosal immune activation. WIRS is one form of emotional stress that has been widely used as a method to make the animal model of gastric mucosal lesions. Stress ulceration is a common disease that was widely used to research the mechanism of acute gastric ulcerations (Rooks et al. 2014; Uramoto et al. 1990). The technique involves restraining an animal in one small fitted cage and immersing it in 23 °C water to the xiphoid level (Ohta et al. 2009).The hypothalamic–pituitary–adrenal axis and the sympatho–adrenal–medullary systems are the main pathways that cause the release of corticosterone, noradrenaline and adrenaline (Pignatelli et al. 1998) and influence the composition of the microbiota. Various acute and chronic stresses lead to the hyper function of the hypothalamic–pituitary–adrenal axis, which accounts for an increased concentration of serum CORT (Rooks et al. 2014). Pro-inflammatory cytokines play a detrimental role in the model (Rooks et al. 2014). IL-17 and IFN-γ, which are pro-inflammatory cytokines, are the most widely studied biomarkers that play crucial roles in the host defence against microbial organisms and in the development of inflammatory diseases (Gu et al. 2013). Thus, we selected three important serum metabolic indices, including CORT, IFN-γ and IL-17, to evaluate our mouse model.

We used the Miseq method, which helps us to gain more comprehensive information and analyse the changes of the faecal flora much further; however, this method has its own bias due to the primer design (V3 region targeted) and the DNA extraction method. Previous studies suggest that stress causes many changes of the intestinal physiology, such as a decrease of the intestinal motility and a functional decline of the immune system that sometimes includes changes in nutritional behaviour and ageing and influences the intestinal flora (Bian et al. 2013). In our study, the composition of the faecal flora changed in the WIRS group; in particular, we observed the proportional increase of *Lachnospiraceae*. These data provides us with information that stress impacts the interaction between the different groups (Bian et al. 2013). *Lachnospiraceae* is known to have an impact on lifespan, healthy phenotypes and the immune responses (Maldonado-Contreras et al. 2011). There was an obvious increase in the *Lachnospiraceae,* after stress, which is associated with human diseases, such as ulcerative colitis, Crohn’s and celiac disease.

In addition, *Bactericides* and *Prevotellaceae* are involved in the determination of the mammalian gut microbiota (Bangsgaard Bendtsen et al. 2012). The molecular and cellular mechanisms remain to be elucidated. The faecal microbiota composition could reflect the gut flora of mice. We found that the basic gut bacterial communities were more similar in the two groups via our heat map results of the bacterial communities derived from the 16SrRNA gene based analyses (Rooks et al. 2014); the basic gut bacterial communities were more similar in the two groups. The Venn diagram reflected that the stress group emerged a new species versus the core intestinal microbiota in the control group. We found faecal microbiota differences in the stress group compared with the normal group by using Illumina Miseq from characterising 367,205 sequences of 16S rRNA genes obtained from ten faecal samples. The most striking shift occurred in the *Lachnospiraceae*, whose frequency increased at the beginning of treatment to 16.20 and 32.41% after the stress treatments. Several publications have introduced that the structural shift of the gut microbiota is associated with human diseases, such as ulcerative colitis, Crohn’s and celiac disease. The faecal flora of the stress had a significant depletion of butyrate-producing bacteria. Especially in after gastric injury, changes in the gut microbiota play as an important role (Rooks et al. 2014).

In conclusion, this study demonstrates that the WIRS model was successful as assessed by behavioural changes, the levels of CORT, IFN-γ, IL-17 and the gastric mucosal injury. The most interesting result was the shift of *Lachnospiraceae* after stress. These changes were associated with stress and affected the balance of the normal intestinal flora. To reveal more details of the stress-induced microbiota shifts, we need more mechanistic research proving our microbiome-wide association study. We think that the above results will improve our understanding of biliary tract microbiota. In addition, these data will be helpful for studies of bacteria-related gallstone pathogenesis and antibiotic therapies.

## Additional file

**Additional file 1.** Additional Figures.

## Abbreviations

WIRS: water immersion restraint stress
CORT: corticosterone
IFN-γ: interferon-γ
IL-17: interleukin-17
PCR: polymerase chain reaction
HE: hematoxylin-eosin
SRA: sequence reads archive
OUT: operational taxonomic units

## References

[CR1] Amato KR, Yeoman CJ, Kent A, Righini N, Carbonero F, Estrada A, Gaskins HR, Stumpf RM, Yildirim S, Torralba M, Gillis M, Wilson BA, Nelson KE, White BA, Leigh SR (2013). Habitat degradation impacts black howler monkey (*Alouatta pigra*) gastrointestinal microbiomes. ISME J.

[CR02] Bangsgaard Bendtsen KM, Krych L, Sørensen DB, Pang W, Nielsen DS (2012). Gut microbiota composition is correlated to grid floor induced stress and behavior in the BALB/c mouse. PLoS ONE.

[CR2] Bian G, Ma L, Su Y, Zhu W (2013). The microbial community in the feces of the white rhinoceros (*Ceratotherium simum*) as determined by barcoded pyrosequencing analysis. PLoS ONE.

[CR3] Blossom SJ, Melnyk SB, Li M, Wessinger WD, Cooney CA (2016). Inflammatory and oxidative stress-related effects associated with neurotoxicity are maintained after exclusively prenatal trichloroethylene exposure. Neurotoxicology.

[CR4] Gu C, Wu L, Li X (2013). IL-17 family: cytokines, receptors and signaling. Cytokine.

[CR5] Guo G, Jia KR, Shi Y, Liu XF, Liu KY, Qi W, Guo Y, Zhang WJ, Wang T, Xiao B, Zou QM (2009). Psychological stress enhances the colonization of the stomach by *Helicobacter pylori* in the BALB/c mouse. Stress.

[CR6] Hsiao A, Ahmed AM, Subramanian S, Griffin NW, Drewry LL, Petri WA, Haque R, Ahmed T, Gordon JI (2014). Members of the human gut microbiota involved in recovery from Vibrio cholerae infection. Nature.

[CR7] Jaggi AS, Bhatia N, Kumar N, Singh N, Anand P, Dhawan R (2011). A review on animal models for screening potential anti-stress agents. Neurol Sci.

[CR8] Kallus SJ, Brandt LJ (2012). The intestinal microbiota and obesity. J Clin Gastroenterol.

[CR09] Maldonado-Contreras A, Goldfarb KC, Godoy-Vitorino F, Karaoz U, Contreras M, Blaser MJ, Brodie EL, Dominguez-Bello MG (2011). Structure of the human gastric bacterial community in relation to Helicobacter pylori status. ISME J.

[CR9] Mohd Fahami NA, Ibrahim IA, Kamisah Y, Mohd Ismail N (2012). Palm vitamin E reduces catecholamines, xanthine oxidase activity and gastric lesions in rats exposed to water-immersion restraint stress. BMC Gastroenterol.

[CR10] Ohta Y, Kaida S, Chiba S, Tada M, Teruya A, Imai Y, Kawanishi M (2009). Involvement of oxidative stress in increases in the serum levels of various enzymes and components in rats with water-immersion restraint stress. J Clin Biochem Nutr.

[CR11] Peerzada KJ, Faridi AH, Sharma L, Bhardwaj SC, Satti NK, Shashi B, Tasduq SA (2016). Acteoside-mediates chemoprevention of experimental liver carcinogenesis through STAT-3 regulated oxidative stress and apoptosis. Environ Toxicol.

[CR12] Pignatelli D, Magalhaes MM, Magalhaes MC (1998). Direct effects of stress on adrenocortical function. Horm Metab Res.

[CR13] Rooks MG, Veiga P, Wardwell-Scott LH, Tickle T, Segata N, Michaud M, Gallini CA, Beal C, van Hylckama-Vlieg JET, Ballal SA, Morgan XC, Glickman JN, Gevers D, Huttenhower C, Garrett WS (2014). Gut microbiome composition and function in experimental colitis during active disease and treatment-induced remission. ISME J.

[CR14] Uramoto H, Ohno T, Ishihara T (1990). Gastric mucosal protection induced by restraint and water-immersion stress in rats. Jpn J Pharmacol.

[CR15] Yan Z, Jiang H, Cai H, Zhou Y, Krumholz LR (2015). Complex interactions between the macrophyte *Acorus Calamus* and microbial fuel cells during pyrene and benzo [a] pyrene degradation in sediments. Sci Rep.

[CR16] Zheng J, Xiao X, Zhang Q, Yu M, Xu J, Qi C, Wang T (2016). The programming effects of nutrition-induced catch-up growth on gut microbiota and metabolic diseases in adult mice. Microbiol Open.

