# Supplementary material for: Lachnospiraceae shift in the microbial community of mice faecal sample effects on water immersion restraint stress
Source: AMB Express. 2017 Apr 17;7:82. doi: 10.1186/s13568-017-0383-4 (PMC5393979; doi:10.1186/s13568-017-0383-4)
Supplement: Supplementary file 1 — Additional file 1. Additional Figures. [file 13568_2017_383_MOESM1_ESM.doc]

**Supporting Information**

# *Lachnospiraceae* shift in the microbial community of mice faecal sample effects on water immersion restraint stress

**Shiyin Li ^a,b†^****, Zelin Wang^a†^, Yun Yang^a^, Sha Yang^a^, Chenchen Yao^a^, Kaiyun Liu^a^, Sixin Cui^a^, Quanming Zou^a^, Hongwu Sun^a*^, Gang Guo^a^****^*^**

(a. National Engineering Research Center of Immunological Products, Department of Microbiology and Biochemical Pharmacy, College of Pharmacy, Third Military Medical University of Chinese PLA , Chongqing ,400038, P.R. China; b. Department of Epidemic Prevention, Hospital of Troop 66325, PLA, Beijing, 102202, P.R. China)

**Correspondence authors**: Hongwu Sun and Gang Guo.

National Engineering Research Center for Immunological Products & Department of Microbiology and Biochemical Pharmacy, College of Pharmacy, Third Military Medical University of Chinese PLA, 30 Gaotanyan Street, Shapingba District, Chongqing, 400038, China

**Tel**: +86-023-68752377; **Fax:** +86-023-68752377

Email: Hongwu Sun, [sunhongwu2001@163.com](mailto:sunhongwu2001@163.com); Gang Guo, guogang7001@163.com

**^†^** These authors contributed equally to this work.

**Fig.S1 HE stains of gastric mucosal tissue after stress**

**
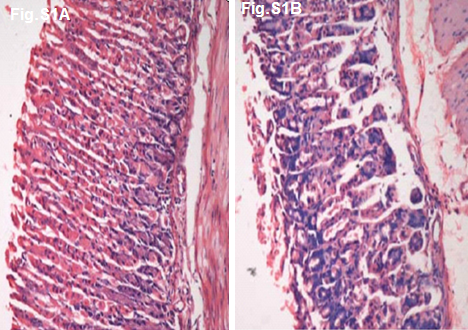
**

Notes: Fig.S1A is gastric mucosal tissue of normal group; Fig.S1B is gastric mucosal tissue of stress group (×200).

**Fig. S2. Rarefaction curve of two group mice: (0.97)**


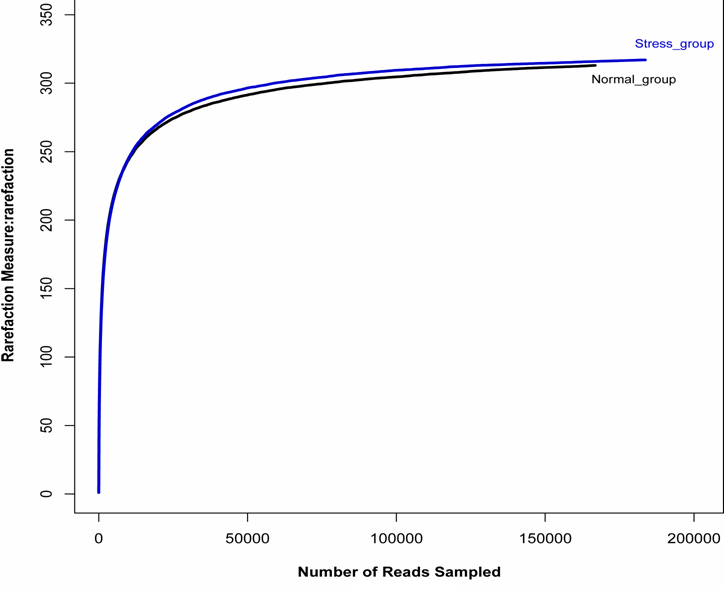


**Fig. S3. Shannon Index of two group mice: (0.97)**


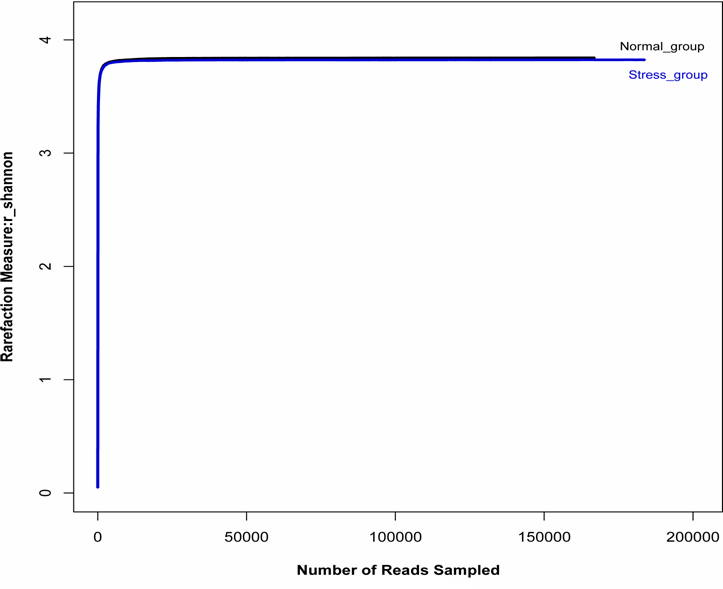


**Fig. S4.Rank Abundance Distribution curve of two group mice**

**
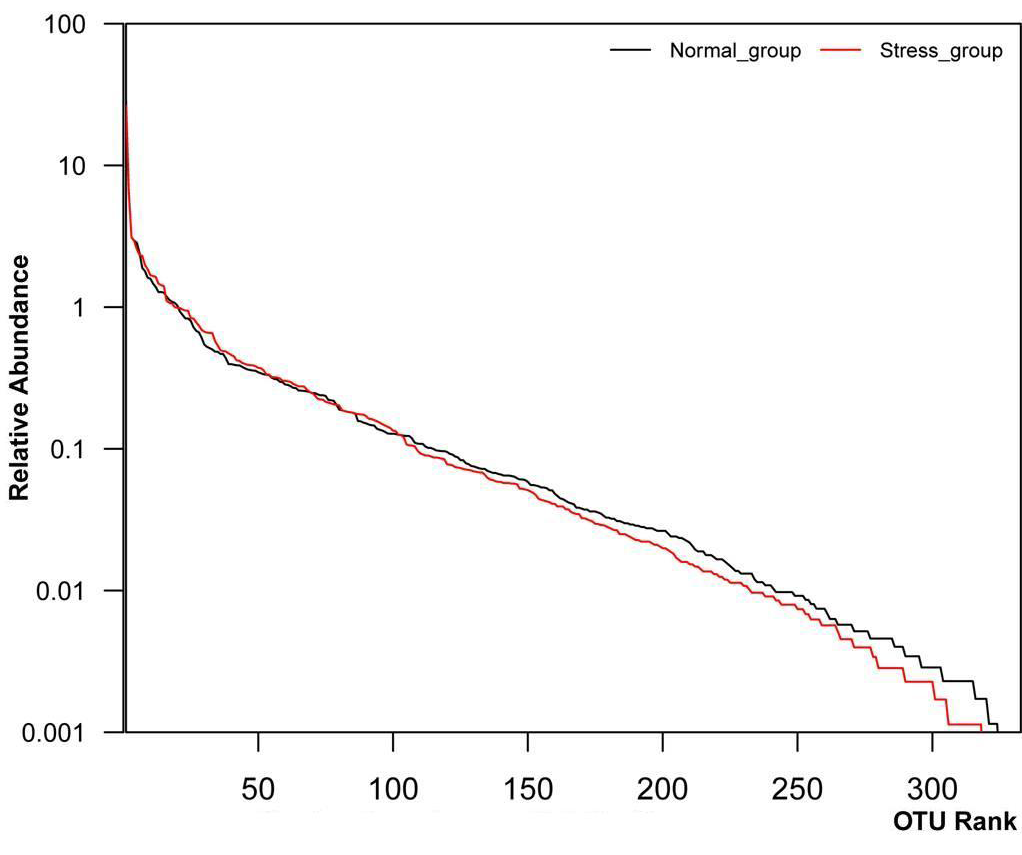
**

**Fig. S5. Specaccum accumulation curve of two group mice**

**
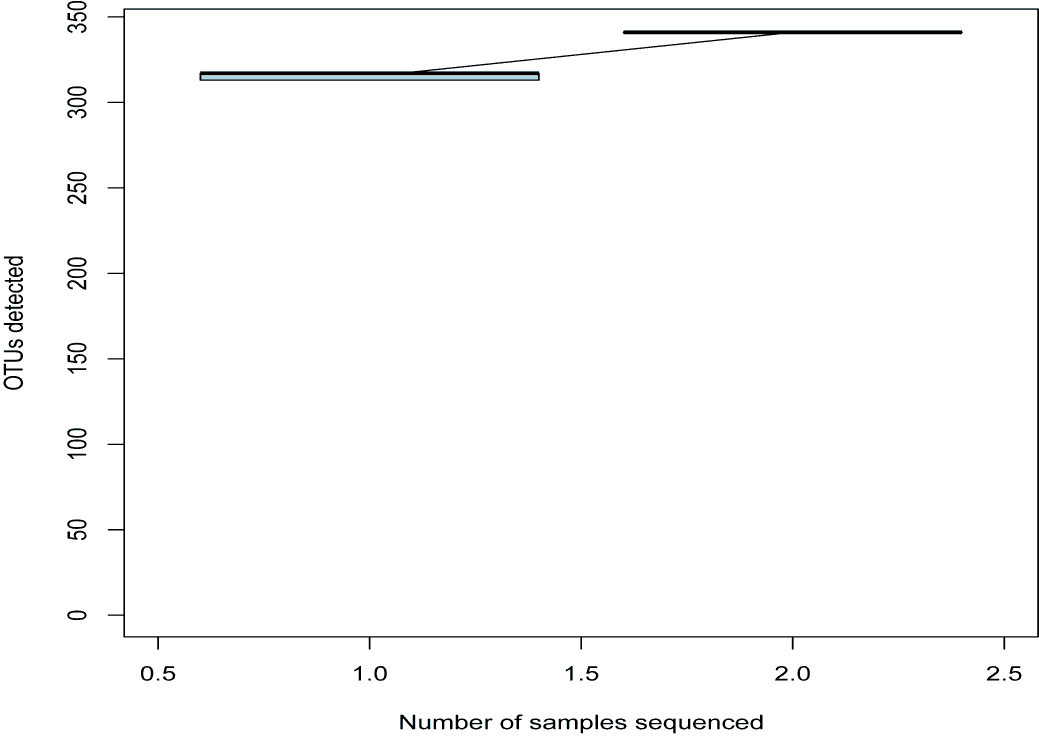
**

**Fig.S6. Heatmap of all mice at family level**


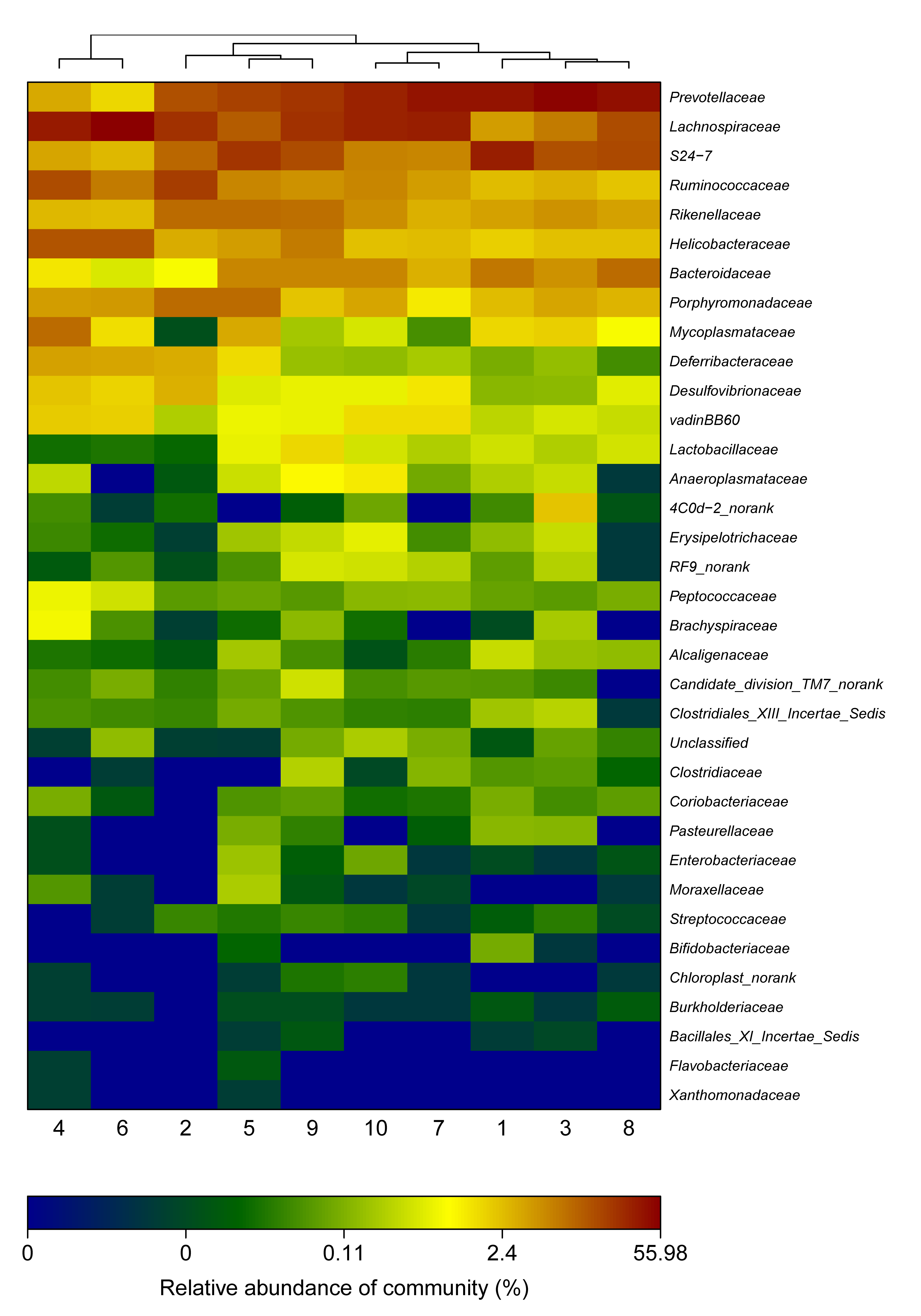


**Note**: Normal mice are from No.1 to No.5. Stress mice are from No.6 to No.10

**Fig.S7. Heatmap of all mice at genus level**


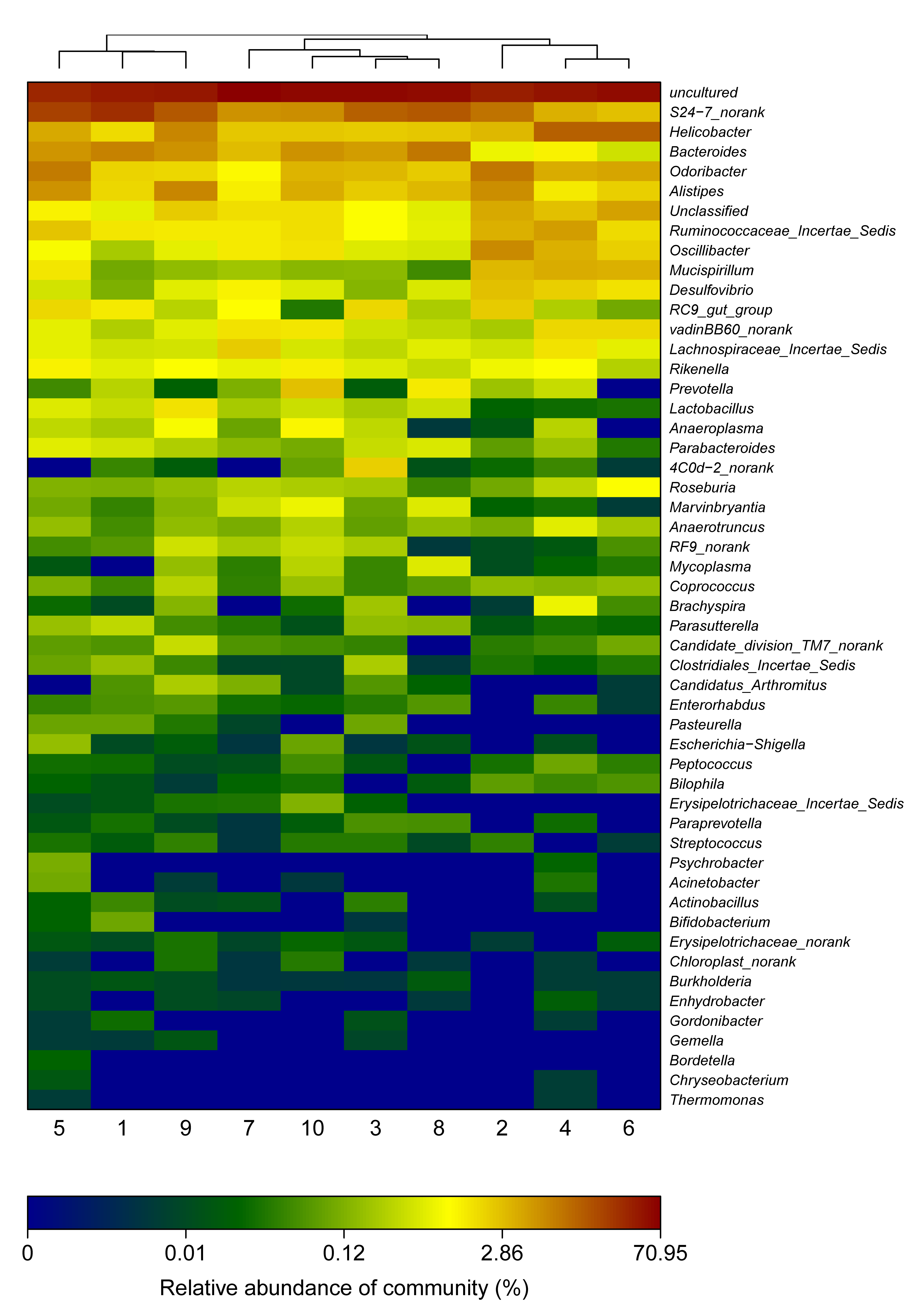


**Note**: Normal mice are from No.1 to No.5. Stress mice are from No.6 to No.10
